# Human leukocyte antigen-dependent colonization of *Lactobacillus* in the early-life gut

**DOI:** 10.3389/frmbi.2023.1192773

**Published:** 2023-09-29

**Authors:** Meghan A. Berryman, Eric W. Triplett, Johnny Ludvigsson

**Affiliations:** ^1^ Department of Microbiology and Cell Science, University of Florida, Gainesville, FL, United States; ^2^ Crown Princess Victoria Children’s Hospital and Division of Pediatrics, Department of Biomedical and Clinical Sciences, Linköping University, Linköping, Sweden

**Keywords:** *Lactobacillus*, ABIS, microbiome, probiotic, type 1 diabetes, celiac disease, DR15-DQ6, DR5-DQ7

## Abstract

To determine the importance of *Lactobacillus* in shaping the human gut microbiome, the microbial composition of stools from 1,602 children between the ages of 0.3 months and 37.2 months was analyzed in a general population cohort in the All Babies in Southeast Sweden study. *Lactobacillus* colonized only 32% of the total pediatric population at an average relative abundance of 0.29%. *Lactobacillus* was age-dependent, decreasing in prevalence and relative abundance over time. The main determining factor for *Lactobacillus* colonization was whether the individual was actively breastfeeding. Following cessation of breastfeeding, *Lactobacillus* prevalence rapidly declined. However, within the actively breastfeeding cohort, 45.6% of the population remained uncolonized by *Lactobacillus*. The presence versus absence of *Lactobacillus* was determined to be human leukocyte antigen (HLA) dependent. Individuals with HLA DR15-DQ6.2 were 3.4 times more likely to be colonized by *Lactobacillus* than those without the haplotype, and those with HLA DR5-DQ7 were more likely to have zero *Lactobacillus* despite actively breastfeeding. These results suggest that HLA genetics should be considered when designing *Lactobacillus*-based probiotics.

## 1 Introduction

Over a century ago, Metchnikoff espoused widely his observations that lactic acid fermenting microbes help prevent intestinal malady, and communities that regularly consumed fermented foods had extended longevity (Metchnikoff, 1908). Today, the study of lactic acid fermenting microbes is substantially driven by the probiotic industry—the global probiotics market size is expected to reach over $100 billion by 2030—and, of the vast selection of probiotic supplements available, *Lactobacillus* and *Bifidobacterium* are the most common (Probiotics: What You Need To Know, 2019; Probiotics Market Size | Industry Report, 2021–2030, 2022). Despite the numerous probiotic research studies and clinical trials published, results are frequently conflicting or inconclusive (Floch et al., 2015; Suez et al., 2019; Berryman et al., 2022). We previously showed in a meta-analysis of probiotic clinical trials for the treatment of autoimmune diseases that 48 of the 65 measurable results from 19 trials were not significant (Berryman et al., 2022). Of the 50 genera used in the examined trials, *Lactobacillus* was included in 42% of the supplementations and *Bifidobacterium* was included in 40%. Despite the probiotic industry’s reliance on *Lactobacillus*, the importance and role that *Lactobacillus* plays in ameliorating dysbiosis remains unclear.

Inconclusive or non-significant results do not mean that the study of potential probiotic therapies is not valuable. Microbial dysbiosis has been associated with autoimmune diseases, neurologic diseases, and cardiovascular diseases (Angelakis et al., 2012; Carding et al., 2015; Russell et al., 2019; Milletich et al., 2022; Kundu et al., 2023; Park et al., 2023). Specifically, *Lactobacillus* enrichment and depletion have been seen in Crohn’s disease, rheumatoid arthritis, obesity, type 2 diabetes, irritable bowel syndrome, type 1 diabetes, HIV, and multiple sclerosis (Heeney et al., 2018). *Lactobacillus* is a commensal bacterium inhabiting humans from birth and has been isolated from the oral cavity, gastrointestinal tract, skin, and vagina (Chu et al., 2017). While *Lactobacillus* can dominate the vaginal microbiome, it makes up ≤ 1% of the adult human gut bacteria (Chu et al., 2017; Heeney et al., 2018).

Since previous studies of both adult and infant populations show that the average relative abundance of *Lactobacillus* in the gut of healthy individuals is quite low (Rossi et al., 2016; Almonacid et al., 2017; Chu et al., 2017; Beck et al., 2022), the objective of this study was to understand levels of *Lactobacillus* in early gut microbiome development and further investigate why a large percentage of the population is not colonized by the genus. We analyzed the microbial composition of stools at different ages from a large general population cohort of children to determine the genus’ colonization patterns over time with respect to its microbial cohabitants. The prevalence of *Lactobacillus* in this population is remarkably low, even with a detection limit of 0.0077%. We hypothesized that *Lactobacillus* may be only a minor player in the human gut microbiome starting in infancy.

## 2 Results

### 2.1 Cohort description

This study analyzed the V3-V4 region of 16S rRNA in stool samples from the prospective, general-population cohort of the All Babies in Southeast Sweden (ABIS) study, which consisted of 17,055 children born between 1 October 1997 and 1 October 1999 in southeast Sweden (Ludvigsson et al., 2001). Biological specimens were longitudinally collected from birth to 13 years of age, creating a biobank of stool, urine, blood, and hair. Metadata was acquired in the form of regular questionnaires, first-year diaries, and human leukocyte antigen (HLA) genotyping. Parental report questionnaires and diaries included information about delivery mode, infection history, antibiotic use, duration of breastfeeding, introduction to and frequency of certain food consumption, living conditions, and additional environmental factors. Stool samples were analyzed from 1,602 children between the ages of 0.3 months and 37.2 months, with an average age of collection of 11.94 months (**Supplementary Table 1**). This analysis included one stool sample from each child, with a total of 1,602 samples analyzed in a between-subjects design. The average read count for the 1,602 samples was 72,113, the minimum read count was 13,183, and the maximum read count was 623,767. To normalize the data, the read counts for the 1,602 samples analyzed were rarefied to the minimum read count of 13,183. Total abundance as reads per gram was calculated by multiplying the relative abundance and copies of 16S rRNA per gram of stool found from qPCR (Jian et al., 2020).

### 2.2 *Lactobacillus* does not colonize the majority of pediatric guts

**Figure 1:**
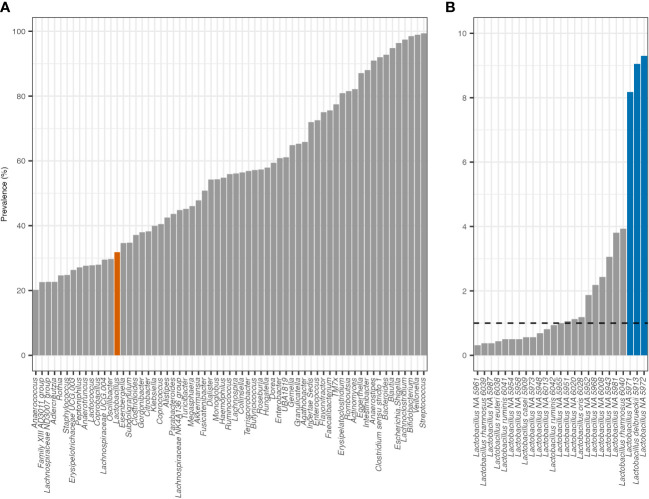
Prevalence of top 30% genera found and all *Lactobacillus* ASVs. **(A)** *Lactobacillus* is highlighted in orange at 31.8% prevalence. **(B)** One percent prevalence is indicated by a dashed line. ASVs over 5% prevalent are highlighted in blue.

Upon analysis of the total population cohort, 1,602 children aged 0.3 months to 37.2 months at stool collection, it was determined that *Lactobacillus* did not colonize the majority of the pediatric population (**Table 1**). An average total abundance of 224,378 (± 4,007,910; min: 0; max: 156,941,755) *Lactobacillus* reads per gram of stool were detected, with an average relative abundance of 0.29% (± 2.3%; min: 0%; max: 74.5%) in the total population. Zero *Lactobacillus* reads were detected in 68.2% of the total population. For comparison, the average total abundance of *Bifidobacterium* was 139,896,70 (± 95,585,095; min: 0; max: 2.4e+09) reads per gram of stool, with an average relative abundance of 16.6% (± 18.8%; min: 0%; max: 98.9%) and 98.5% prevalence. *Lactobacillus* ranked 50th in prevalence out of the 205 genera found and populated 31.8% of the entire ABIS cohort studied here (**Figure 1A**).

**Table 1:** Prevalence of *Lactobacillus* absence vs. presence in total and subset cohorts.

		*Lactobacillus*	
	Cohort size	Absence	Presence
**Total population**	1,602 (100%)	1,092 (68.2%)	510 (31.8%)
**Age: 0–3 months**	16 (9.9%)	11 (68.7%)	5 (31.3%)
**Age: 3–6 months**	88 (5.5%)	40 (45.5%)	48 (54.5%)
**Age: 6–12 months**	543 (33.9%)	369 (68%)	174 (32.0%)
**Age: 12–18 months**	854 (53.3%)	610 (71.4%)	244 (28.6%)
**Age: >18 months**	27 (1.7%)	20 (74.1%)	7 (25.9%)
**Actively breastfeeding**	83 (5.1%)	37 (44.6%)	46 (55.4%)
**Not breastfeeding**	1,273 (79.4%)	898 (70.5%)	375 (29.5%)
**DR15-DQ6.2 present**	18 (1.1%)	4 (22.2%)	14 (77.8%)
**DR5-DQ7 present**	12 (0.7%)	10 (83.3%)	2 (16.7%)
**DR8-DQ4 present**	6 (0.4%)	5 (83.3%)	1 (16.7%)

Active breastfeeding: diet included breastmilk at the time of stool sample collection. Not breastfeeding: diet did not currently include breastmilk at the time of stool sample collection.

Within the genus, 25 *Lactobacillus* amplicon sequence variants (ASVs), each with at least one nucleotide difference, were found within the total population. Every *Lactobacillus* ASV was detected in a small percentage of the cohort. Thirteen ASVs were in fewer than 1% of the population and only three were in more than 5% of the population, at 9.3%, 9.1%, and 8.2% prevalence, respectively (**Figure 1B**). The top three ASVs in more than 5% of the population were identified using the SILVA_v138 database as *Lactobacillus* NA 5972, *Lactobacillus delbrueckii* 5913, and *Lactobacillus* NA 5971. The 16S sequences from each ASV were individually searched with NCBI BLAST and verified or identified with 100% query coverage and ≥ 99.7% identity. The top three ASVs were identified as *Lacticaseibacillus paracasei, Lactobacillus delbrueckii*, and *Lacticaseibacillus rhamnosus* (**Supplementary Table 2**). The frequency of possessing multiple *Lactobacillus* ASVs was low, with only 11.8% of the population colonized by more than one *Lactobacillus* ASV—321 children (20.0%) had one ASV, 96 (5.9%) had two ASVs, 44 (3.7%) had three ASVs, 25 (1.6%) had four ASVs, 15 (0.9%) had five ASVs, six (0.4%) had six ASVs, and three (0.2%) had seven ASVs. No individual was colonized by more than seven *Lactobacillus* ASVs.

### 2.3 *Lactobacillus* is age-dependent and associated with breastfeeding

**Figure 2:**
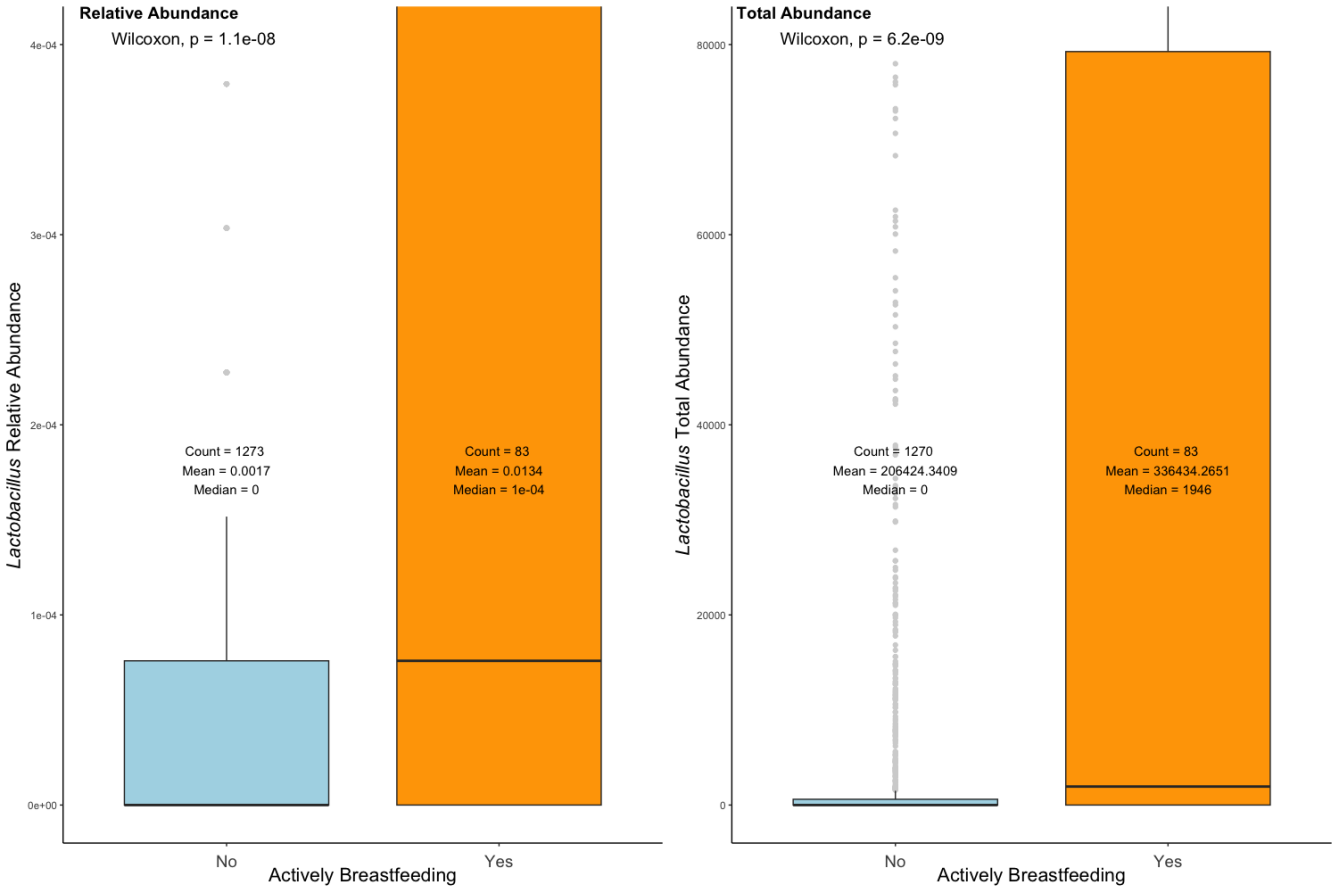
Relative abundance and total abundance of *Lactobacillus* in the actively breastfeeding and not breastfeeding cohorts. Active breastfeeding: yes—diet included breastmilk at the time of stool sample collection; no—diet did not currently include breastmilk at the time of stool sample collection. The significance was determined with the Wilcoxon rank sum test.

In the total population, *Lactobacillus* relative abundance and total abundance were negatively correlated with increasing age calculated with Spearman’s rank correlation (RA: rho = −0.096, *p* = 1.6e-4; TA: rho = −0.10, *p* = 8.9e-5). In addition, whether a child was actively breastfeeding when their stool sample was provided was the only variable significantly associated with both *Lactobacillus* relative abundance and/or total abundance when adjusted for false discovery rates using the Benjamini–Hochberg method (Kruskal–Wallis: p_adj_ = 2.4e-17 and p_adj_ = 4.2e-18, respectively) (**Figure 2**). An individual was categorized as actively breastfeeding if their diet included breastmilk, either exclusively or supplementally, at the time of stool sample collection. An individual was categorized as not breastfeeding if their diet did not include breastmilk at the time of stool sample collection.

**Figure 3:**
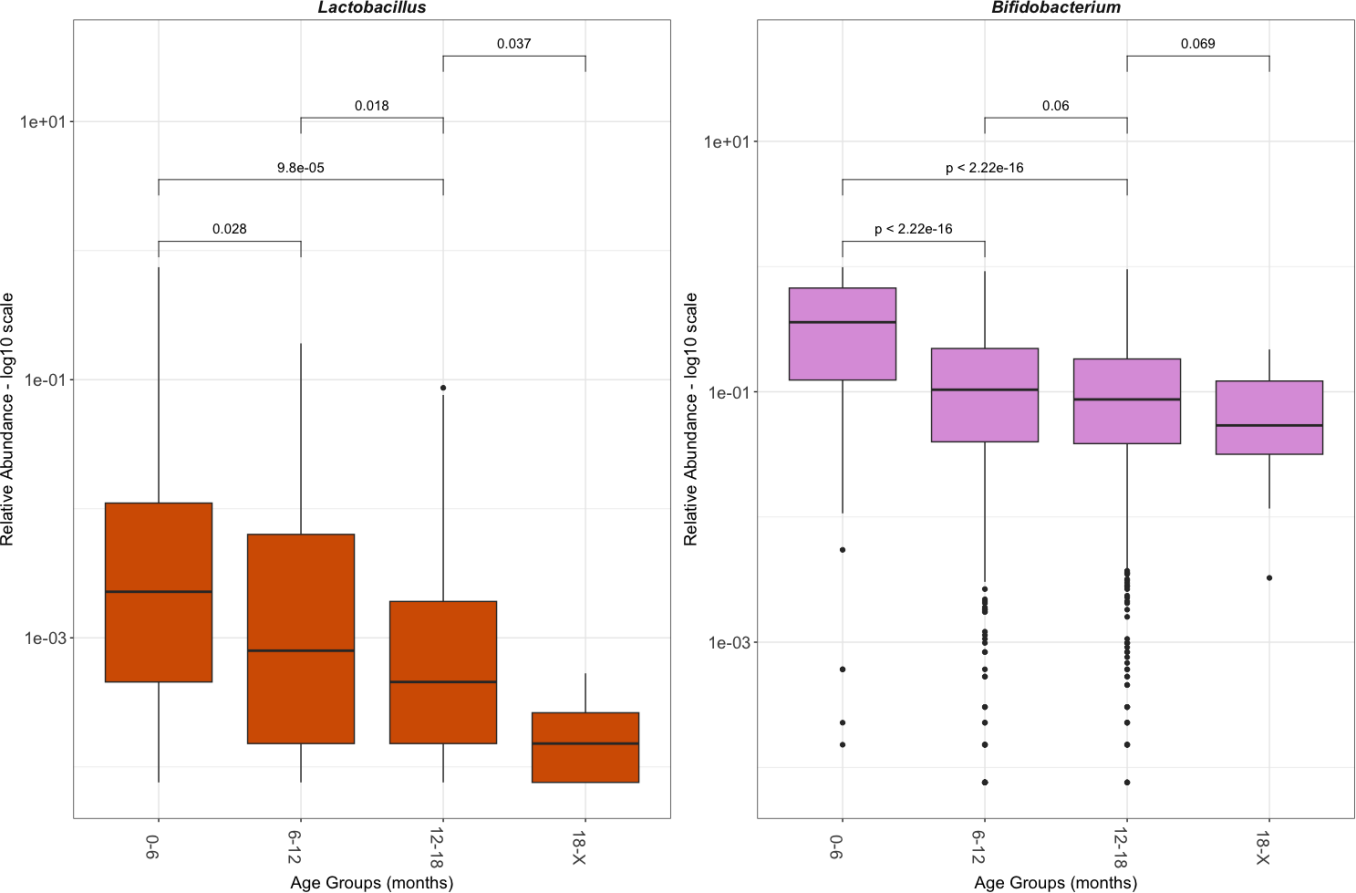
Relative abundance of *Lactobacillus* and *Bifidobacterium* by age group. Relative abundance is shown in a log 10 scale. This reflects only those with *Lactobacillus* or *Bifidobacterium*, excluding the population without the genus. The significance was determined with a Wilcoxon rank sum test.

Further investigation into the defining features of those who were colonized by *Lactobacillus*, referred to here as the *Lactobacillus* presence cohort (LPC), was performed. The average total abundance of *Lactobacillus* in the LPC was 705,820 (± 7,089,251; min: 4; max: 156,941,755) reads per gram of stool, with 0.92% (± 3.9%; min: 0.0075%; max: 74.5%) average relative abundance. As with the total population in the LPC, *Lactobacillus* relative abundance decreased significantly with age (**Figure 3**). In the 0 months to 6 months LPC, the mean relative abundance of *Lactobacillus* was 2.3% (± 10.7%). In the 6 months to 12 months LPC, the mean relative abundance of *Lactobacillus* was 0.79% (± 2.0%). In the 12 months to 18 months LPC, the mean relative abundance of *Lactobacillus* was 0.51% (± 1.4%). In the over 18 months LPC, the mean relative abundance of *Lactobacillus* was 0.02% (± 0.01%). The relative abundance of *Bifidobacterium* was shown over time for comparison: it significantly declines after 6 months but remains steady. *Lactobacillus* continued to significantly decrease as age increased. Through a chi-squared test of independence, *Lactobacillus* presence versus absence was most dependent on active breastfeeding (p_adj_ = 0.028) (**Supplementary Table 3**).

**Figure 4:**
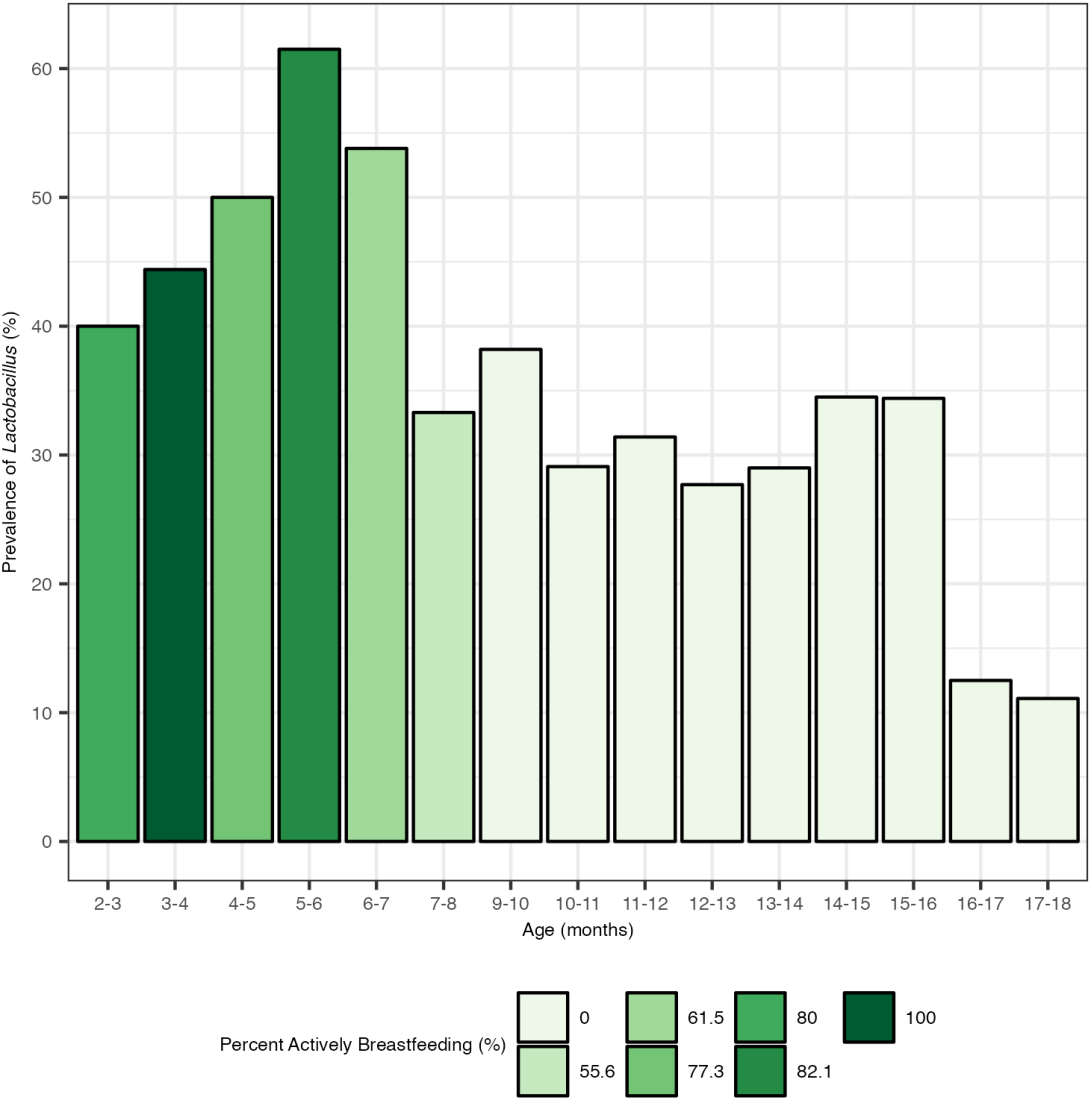
Prevalence of *Lactobacillus* by month and percentage of children actively breastfeeding. Actively breastfeeding is defined here as an individual whose diet included breastmilk at the time of stool sample collection. There were no stool samples collected at age 8–9 months.

The prevalence of *Lactobacillus* within the total population increased during the period from birth to 6 months, a time period when the majority of the population was actively breastfeeding (**Figure 4**). Despite the majority of the population continuing to receive breastmilk up to 9 months, the prevalence of *Lactobacillus* colonization in the total population began to decline after 6 months and dropped starkly after 7 months. The 5 months to 6 months age group had the highest prevalence of *Lactobacillus* colonization at 61.5%. After 6 months, the prevalence of *Lactobacillus* colonization dropped from 53.8% at age 6–7 months to 33.3% at age 7–8 months, and 29.1% by 10–11 months. By 17–18 months, *Lactobacillus* colonization was at 11.1% prevalence.

### 2.4 Human leukocyte antigen associated with *Lactobacillus* presence in actively breastfeeding cohort

While actively breastfeeding was a major determinant of *Lactobacillus* presence, relative abundance, and total abundance, only 55.4% of the total actively breastfeeding portion of the cohort (ABC) was colonized by *Lactobacillus* (**Table 1**). Since 45.6% of the ABC remain uncolonized by *Lactobacillus*, statistical analysis was performed to investigate which variables impact whether an actively breastfeeding child is colonized or not. It was determined that microbial factors did not influence *Lactobacillus* colonization in the ABC. No other genera relative abundances or total abundances were significantly associated with *Lactobacillus* presence or absence, when Wilcoxon rank-sum tests were performed and *p*-values were adjusted for false discovery rate (data not shown). There were also no strong correlations between *Lactobacillus* relative abundance and other genera with Spearman’s rank correlations. Subsequently, it was determined that additional environmental factors did not affect the colonization of *Lactobacillus* in the ABC. Chi-squared tests and a Kruskal–Wallis one-way analysis of variance of the environmental factors in parental report questionnaires and diaries showed no significant association with *Lactobacillus* presence versus absence or relative abundance (data not shown).

**Figure 5:**
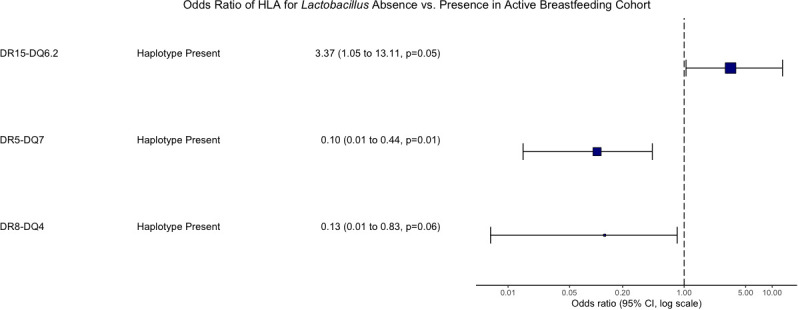
Odds ratio of HLA associated with *Lactobacillus* presence vs. absence in the active breastfeeding Cohort. An odds ratio of greater than 1 means those with the haplotype have greater odds of having *Lactobacillus*. An odds ratio less than 1 means those with the haplotype have greater odds of not having *Lactobacillus.* Only p-values < 0.1 are shown.

Human leukocyte antigen genetics was the only factor tested that showed a significant relationship with whether an actively breastfeeding infant was colonized by *Lactobacillus*. *Lactobacillus* presence was determined to be dependent on HLA haplotypes DR5-DQ7 (*p* = 0.0045), DR15-DQ6.2 (p = 0.048), and DR8-DQ4 (p = 0.078) through a chi-squared test of independence. An odds ratio analysis indicated that individuals with HLA DR15-DQ6.2 were 3.4 times more likely to be colonized by *Lactobacillus* than those without that haplotype. Individuals with HLA DR5-DQ7 were more likely to not be colonized by *Lactobacillus* despite actively breastfeeding (**Figure 5**).

## 3 Discussion

Our analysis of 1,602 pediatric microbiomes over time reaffirms that *Lactobacillus* is not a major component of the gut after 6 months of age. At an average age of 11.94 months, nearly 70% of the total population was not colonized by *Lactobacillus* at all. Of those who were colonized by *Lactobacillus*, the relative abundance decreased steadily over time. For most of the population, *Lactobacillus* did not remain in the gut following the cessation of breastfeeding. Breastfeeding was the major determinant of whether an individual was colonized by *Lactobacillus* or not and the relative and total abundances an individual had. Controlling for active breastfeeding revealed HLA associations that had previously been masked. Individuals with HLA DR15-DQ6.2 were 3.4 times more likely to be colonized by *Lactobacillus* than those without the haplotype, and those with HLA DR5-DQ7 were more likely to have zero *Lactobacillus* despite actively breastfeeding.

Our results confirm that the low amounts of *Lactobacillus* colonizing the human gut start in infancy. Previous studies of both adult and infant populations show that the average relative abundance of *Lactobacillus* in healthy individuals without supplementation is ~0.3%, which matches our finding of the 0.29% average relative abundance in the pediatric population studied here (Rossi et al., 2016; Almonacid et al., 2017; Chu et al., 2017; Beck et al., 2022). While a general population study of human adults found 43 *Lactobacillus* strains consistently residing in the gut (Rossi et al., 2016), this study observed only 25 *Lactobacillus* ASVs within the pediatric population, which may be a result of exposure due to age.

*Lactobacillus* and *Bifidobacterium* are predominant composites of the human breastmilk microbiome (Łubiech and Twarużek, 2020). It is unsurprising that both *Lactobacillus* and *Bifidobacterium* levels are higher up to 6 months of age, which is when the cohort was primarily breastfeeding. However, at our levels of detection, *Lactobacillus* did not appear to remain in most guts following the cessation of breastfeeding. *Lactobacillus* abundance and prevalence increased at 3 months and declined after 6 months as the population began to cut back on breastfeeding. The persistent decline in *Lactobacillus* relative abundance over time was not observed in the other breastfeeding-associated genus, *Bifidobacterium*. The lack of persistent *Lactobacillus* after supplementation was consistent with a recent infant probiotic study that showed that following treatment with *Bifidobacterium-* and *Lactobacillus*-based probiotics, some *Bifidobacterium* persisted but no *Lactobacillus* persisted in the infant gut (Beck et al., 2022). *Bifidobacterium* was noticeably more abundant than *Lactobacillus* in the cohort studied here, which is supported by previous studies (Vatanen et al., 2018).

When controlling for active breastfeeding, an association with interesting implications was revealed. Individuals with HLA DR15-DQ6.2 were more likely to be colonized by *Lactobacillus* and those with HLA DR5-DQ7 were more likely to have zero *Lactobacillus* despite actively breastfeeding. The influence that active supplementation of *Lactobacillus* through breastfeeding had on the gut microbiome composition likely masked the influences of genetics during initial investigations. Still, almost half of the actively breastfeeding population in this study was not colonized by *Lactobacillus* at all. The determining factor for this lack of colonization was possessing HLA DR5-DQ7. To our knowledge, the negative association between HLA DR5-DQ7 and *Lactobacillus* has not been previously reported. However, DR5-DQ7 is the third most abundant haplotype in celiac disease patients, often seen in a heterozygote pair with DR7-DQ2 (Tinto et al., 2015; Pisapia et al., 2020). Together with evidence that *Lactobacillus* is seen in lower abundance in celiac disease than in healthy control patients (Lorenzo Pisarello et al., 2015), this suggests that HLA is playing an important role in shaping the microbiome. HLA DR15-DQ6.2 is a known protective allele against type 1 diabetes (Thomas et al., 2021), and its link here with *Lactobacillus* further emphasizes the need to carefully consider autoimmune-associated genetics when studying probiotic bacteria.

*Lactobacillus rhamnosus* was among the highest *Lactobacillus* ASV detected in this study. Interestingly, *L. rhamnosus* is higher in healthy controls and may be protective against islet autoimmunity (Vatanen et al., 2018). However, *Lactobacillus* was included in 42% of the supplementations in 19 probiotic clinical trials for the treatment of autoimmune disease over the past 10 years, and the majority of the results were not significant. Specifically, *L. rhamnosus* was included in all three of the T1D probiotic studies and none of them showed prevention or amelioration of the disease (Berryman et al., 2022). None of these studies controlled for genetics, however. The potential for success may be found if future studies take HLA into consideration. While *Lactobacillus* is arguably poor at colonizing the gut, a previous study concluded that heat-inactivated, non-colonizing *Lactobacillus* strains were sufficient in promoting the regulation of intestinal epithelial barrier function (Singh et al., 2021), suggesting that further analysis needs to be conducted to determine if the low colonization level of *Lactobacillus* is an inhibiting factor when acting as a probiotic.

*Lactobacillus* is a minor player in the human gut microbiome starting in infancy, even during breastfeeding. It does not readily colonize the gut without active and persistent intake. As a potential component of probiotic supplementation, it is important to consider the role HLA may play in promoting or preventing colonization of *Lactobacillus*. If autoimmune risk-associated HLAs are inhibiting the colonization of potentially beneficial bacteria, it could indicate that the individuals who could benefit most from the amelioration of dysbiosis are not being treated efficiently by the probiotics available. Future research is needed to determine the best probiotic formula for high-risk individuals.

## 4 Materials and methods

### 4.1 Sample collection and institutional review board approval

The All Babies in Southeast Sweden (ABIS) study included a prospective general population cohort of 17,055 children born in southeast Sweden between 1 October 1997 and 1 October 1999. Parental consent followed oral, written, and video information about the study. Following parental consent, participating parents completed questionnaires and diaries from birth through the first year of life (Ludvigsson et al., 2001). Collected information included but was not limited to infection history, antibiotic use, duration of breastfeeding, introduction to or frequency of certain food consumption, living conditions, and additional environmental factors. Approval was obtained by the Research Ethics Committees of the Faculty of Health Science at Linköping University, Sweden, Ref. 1997/96,287 and 2003/03-092, and the Medical Faculty of Lund University, Sweden (Dnr 99227, Dnr 99321) as described previously (Russell et al., 2019). The microbial analysis performed at the University of Florida was approved by the University of Florida’s Institutional Review Board as an exempt study IRB201800903.

Pediatric stool samples were collected from 1,756 individual children aged 0.3 months to 37.2 months—the average age of collection was 11.94 months—and processed as previously reported (Russell et al., 2019). Following filtering, this analysis included one stool sample from each child, with a total of 1,602 samples analyzed in a between-subject effect design.

All available HLA genotypes determined from blood spots were included in the analysis. HLA DR-DQ haplotypes of interest here were defined as follows: DR5-DQ7 (DRB1*11-DQA1*05-DQB1*03); DR8-DQ4 (DRB1*08-DQA1*03:03-DQB1*04); and DR15-DQ6.2 (DRB1*15-DQA1*01:02-B1*06:02). Typing was performed with sequence-specific lanthanide-labeled oligonucleotide hybridization (Ilonen et al., 2016).

### 4.2 Processing amplicon sequence variants

DNA extraction, 16S rRNA barcoded PCR, and V3-V4 16S rRNA Illumina sequencing were performed on stool samples collected as previously described (Russell et al., 2019). Universal 16S rRNA primers were used to perform bacterial quantification through quantitative polymerase chain reaction (qPCR) as previously described (Russell et al., 2021). Paired-end reads were merged, primers were removed, and ASV processing was performed as previously described (Milletich et al., 2022). Taxonomy was assigned via the assignTaxonomy() function using the SILVA_v138 database within the R package DADA2 (version 1.26) (Pruesse et al., 2007; Quast et al., 2013; Callahan et al., 2016). Species assignments for Lactobacillus ASVs that were below default minimum boostrapping confidence were determined via NCBI BLAST with a 100% query coverage and ≥ 99.7% identity (**Supplementary Table 2**) (Altschul et al., 1990; Boratyn et al., 2012).

Filtering the original 1,756 children to exclude samples with fewer than 1,000 total reads resulted in the analysis of stool samples from 1,602 children. Filtering to remove ASVs with fewer than five reads using the filter_taxa function from phyloseq (version 1.42.0) resulted in 2,102 ASVs (McMurdie and Holmes, 2013). The average read count for the 1,602 samples analyzed was 72,113, the minimum read count was 13,183, and the maximum read count was 623,767. To normalize the data, the read counts for the 1,602 samples analyzed were rarefied to the minimum read count of 13,183 using rarefy_even_depth() from the phyloseq package. The relative abundance of samples was determined with transform_sample_counts, and the reads/g was calculated by multiplying the relative abundance and copies of 16S rRNA per gram of stool found from qPCR (Jian et al., 2020). ASVs were conglomerated into genera using the tax_glom() function from phyloseq.

### 4.3 Statistical analysis

All packages were run on RStudio (version 2023.03.0 + 386) (Posit team, 2023). Variables impacting binomial beta diversity were tested for using the permutational multivariate analysis of variance (PERMANOVA) test through the adonis() function in the package vegan (version 2.4-6) (Oksanen et al., 2020). Alpha diversity was calculated with the R function plot_richness() in the phyloseq package. Confounding factors of *Lactobacillus* abundance and relative abundance were determined via a non-parametric Kruskal–Wallis rank sum test using the R function kruskal.test(). Genera impacted by the presence or absence of *Lactobacillus* were determined via the non-parametric Wilcoxon rank sum test using the R function wilcox.test(), and *p*-values were corrected for false discovery rates (FDRs) using the Benjamini–Hochberg method using the R function p.adjust().

The R package PIME (version 0.1.0) determined core microbiomes of each cohort (Roesch et al., 2020). The plot_ordination() function constructed the associated principal coordinate analysis (PCoA) plot.

Relative abundances were calculated using the transform_sample_counts function. To calculate total abundance, the relative abundance values were multiplied by the total number of copies of 16s rRNA per gram of stool, as determined through qPCR. Prevalence was defined as the percentage of individuals in the cohort with non-zero abundance of the respective ASV or taxa. Prevalence was calculated using the getPrevalence() function from R package mia (version 1.4.0) (Ernst et al., 2023).

The odds ratio was calculated with the logistic regression model R function glm() and odds.ratio() function from the questionr package (version 0.7.8). The Spearman’s correlation was calculated with the R function cor(). The correlation plot was created using the R package corrplot (version 0.92) (Wei and Simko, 2023). Odds ratios were calculated using the oddsratio() function from the R package epitools (version 10.1) and MedCalc Software Ltd (Aragon et al., 2020; Schoonjans, n.d). The odds ratio plot was created using the or_plot() function from the finalfit package (version 1.0.6) (Harrison et al., 2023). The heatmaps were designed using the R package pheatmap (version 1.0.12) (Kolde, 2019). Boxplots were designed using the R package ggplot2 (version 3.4.0) (Wickham, 2016). The function stat_compare_means() was used to determine the Kruskal–Wallis and Wilcoxon *p*-values. The function ggarrange() was used to arrange each graph into a figure.

## Data availability statement

The original contributions presented in the study are included in the **Supplementary Material** **Data 1** and **Data 2**, further inquiries can be directed to the corresponding author.

## Ethics statement

The studies involving humans were approved by Research Ethics Committees of the Faculty of Health Science at Linköping University, Sweden, Ref. 1997/96287 and 2003/03-092 and the Medical Faculty of Lund University, Sweden (Dnr 99227, Dnr 99321). The studies were conducted in accordance with the local legislation and institutional requirements. Written informed consent for participation in this study was provided by the participants’ legal guardians/next of kin.

## Author contributions

MB developed the concept, performed the primary data analysis, and wrote the paper. ET assisted with concept development and interpretation of data. JL founded and coordinated ABIS, designed the study, and carried out sample collection, storage, and transport. All authors contributed to the article and approved the submitted version.

## References

[B1] Almonacid D. E. Kraal L. Ossandon F. J. Budovskaya Y. V. Cardenas J. P. Bik E. M.. (2017). 16S rRNA gene sequencing and healthy reference ranges for 28 clinically relevant microbial taxa from the human gut microbiome. PloS One 12, e0176555. doi: 10.1371/journal.pone.0176555 28467461 PMC5414997

[B2] Altschul S. F. Gish W. Miller W. Myers E. W. Lipman D. J. (1990). Basic local alignment search tool. J. Mol. Biol. 215, 403–410. doi: 10.1016/S0022-2836(05)80360-2 2231712

[B3] Angelakis E. Armougom F. Million M. Raoult D. (2012). The relationship between gut microbiota and weight gain in humans. Future Microbiol. 7, 91–109. doi: 10.2217/fmb.11.142 22191449

[B4] Aragon T. J. Fay M. P. Wollschlaeger D. Omidpanah A. (2020). epitools: epidemiology tools. Available at: https://CRAN.R-project.org/package=epitools (Accessed December 5, 2022).

[B5] (2022) Probiotics market size | Industry report 2021-2030. Available at: https://www.grandviewresearch.com/industry-analysis/probiotics-market (Accessed March 8, 2023).

[B6] (2019) Probiotics: what you need to know (NCCIH). Available at: https://www.nccih.nih.gov/health/probiotics-what-you-need-to-know (Accessed March 8, 2023).

[B7] Beck L. C. Masi A. C. Young G. R. Vatanen T. Lamb C. A. Smith R.. (2022). Strain-specific impacts of probiotics are a significant driver of gut microbiome development in very preterm infants. Nat. Microbiol. 7, 1525–1535. doi: 10.1038/s41564-022-01213-w 36163498 PMC9519454

[B8] Berryman M. A. Milletich P. L. Petrone J. R. Roesch L. F. W. Ilonen J. Triplett E. W.. (2022). Autoimmune-associated genetics impact probiotic colonization of the infant gut. J. Autoimmun. 133, 102943. doi: 10.1016/j.jaut.2022.102943 36356550

[B9] Boratyn G. M. Schäffer A. A. Agarwala R. Altschul S. F. Lipman D. J. Madden T. L. (2012). Domain enhanced lookup time accelerated BLAST. Biol. Direct 7, 12. doi: 10.1186/1745-6150-7-12 22510480 PMC3438057

[B10] Callahan B. J. McMurdie P. J. Rosen M. J. Han A. W. Johnson A. J. A. Holmes S. P. (2016). DADA2: High resolution sample inference from Illumina amplicon data. Nat. Methods 13, 581–583. doi: 10.1038/nmeth.3869 27214047 PMC4927377

[B11] Carding S. Verbeke K. Vipond D. T. Corfe B. M. Owen L. J. (2015). Dysbiosis of the gut microbiota in disease. Microb. Ecol. Health Dis. 26, 26191. doi: 10.3402/mehd.v26.26191 PMC4315779 25651997

[B12] Chu D. M. Ma J. Prince A. L. Antony K. M. Seferovic M. D. Aagaard K. M. (2017). Maturation of the infant microbiome community structure and function across multiple body sites and in relation to mode of delivery. Nat. Med. 23, 314–326. doi: 10.1038/nm.4272 28112736 PMC5345907

[B13] Ernst F. Shetty S. Borman T. Lahti L. (2023). mia: Microbiome analysis. Available at: https://github.com/microbiome/mia (Accessed December 5, 2022).

[B14] Floch M. H. Walker W. A. Sanders M. E. Nieuwdorp M. Kim A. S. Brenner D. A.. (2015). Recommendations for probiotic use–2015 update: proceedings and consensus opinion. J. Clin. Gastroenterol. 49 Suppl 1, S69–S73. doi: 10.1097/MCG.0000000000000420 26447969

[B15] Harrison E. Drake T. Ots R. (2023) finalfit: Quickly Create Elegant Regression Results Tables and Plots when Modelling. Available at: https://github.com/ewenharrison/finalfit (Accessed July 26, 2023).

[B16] Heeney D. D. Gareau M. G. Marco M. L. (2018). Intestinal *Lactobacillus* in health and disease, a driver or just along for the ride? Curr. Opin. Biotechnol. 49, 140–147. doi: 10.1016/j.copbio.2017.08.004 28866243 PMC5808898

[B17] Ilonen J. Kiviniemi M. Lempainen J. Simell O. Toppari J. Veijola R.. (2016). Genetic susceptibility to type 1 diabetes in childhood – estimation of HLA class II associated disease risk and class II effect in various phases of islet autoimmunity. Pediatr. Diabetes 17, 8–16. doi: 10.1111/pedi.12327 27411431

[B18] Jian C. Luukkonen P. Yki-Järvinen H. Salonen A. Korpela K. (2020). Quantitative PCR provides a simple and accessible method for quantitative microbiota profiling. PloS One 15, e0227285. doi: 10.1371/journal.pone.0227285 31940382 PMC6961887

[B19] Kolde R. (2019). pheatmap: pretty heatmaps. Available at: https://CRAN.R-project.org/package=pheatmap (Accessed July 6, 2022).

[B20] Kundu S. Nayak S. Rakshit D. Singh T. Shukla R. Khatri D. K.. (2023). The microbiome-gut-brain axis in Epilepsy: Pharmacotherapeutic target from bench evidence for potential bedside applications. Eur. J. Neurol. doi: 10.1111/ene.15767 36880679

[B21] Lorenzo Pisarello M. J. Vintiñi E. O. González S. N. Pagani F. Medina M. S. (2015). Decrease in lactobacilli in the intestinal microbiota of celiac children with a gluten-free diet, and selection of potentially probiotic strains. Can. J. Microbiol. 61, 32–37. doi: 10.1139/cjm-2014-0472 25438612

[B22] Łubiech K. Twarużek M. (2020). *Lactobacillus* bacteria in breast milk. Nutrients 12, 3783. doi: 10.3390/nu12123783 33321792 PMC7764098

[B23] Ludvigsson J. Ludvigsson M. Sepa A. (2001). Screening for prediabetes in the general child population: maternal attitude to participation. Pediatr. Diabetes 2, 170–174. doi: 10.1034/j.1399-5448.2001.20405.x 15016182

[B24] McMurdie P. J. Holmes S. (2013). phyloseq: an R package for reproducible interactive analysis and graphics of microbiome census data. PloS One 8, e61217. doi: 10.1371/journal.pone.0061217 23630581 PMC3632530

[B25] Metchnikoff E. (1908). The prolongation of life: optimistic studies (New York, NY: G.P. Putnam’s Sons).

[B26] Milletich P. L. Ahrens A. P. Petrone J. R. Russell J. T. Berryman M. A. Agardh D.. (2022). Gut microbiome markers in subgroups of HLA class II genotyped infants signal future celiac disease in the general population: ABIS study. Front. Microbiol. 12, 920735. doi: 10.3389/fcimb.2022.920735 PMC9357981 35959362

[B27] Oksanen J. Blanchet F. G. Friendly M. Kindt R. Legendre P. McGlinn D.. (2020). vegan: community ecology package. Available at: https://CRAN.R-project.org/package=vegan (Accessed April 6, 2022).

[B28] Park J. M. Lee S. C. Ham C. Kim Y. W. (2023). Effect of probiotic supplementation on gastrointestinal motility, inflammation, motor, non-motor symptoms and mental health in Parkinson’s disease: a meta-analysis of randomized controlled trials. Gut Pathog. 15, 9. doi: 10.1186/s13099-023-00536-1 36879342 PMC9990363

[B29] Pisapia L. Picascia S. Farina F. Barba P. Gianfrani C. Del Pozzo G. (2020). Differential expression of predisposing HLA-DQ2.5 alleles in DR5/DR7 celiac disease patients affects the pathological immune response to gluten. Sci. Rep. 10, 17227. doi: 10.1038/s41598-020-73907-2 33057065 PMC7560598

[B30] Posit team (2023). RStudio: integrated development environment for R. Available at: http://www.posit.co/.

[B31] Pruesse E. Quast C. Knittel K. Fuchs B. M. Ludwig W. Peplies J.. (2007). SILVA: a comprehensive online resource for quality checked and aligned ribosomal RNA sequence data compatible with ARB. Nucleic Acids Res. 35, 7188–7196. doi: 10.1093/nar/gkm864 17947321 PMC2175337

[B32] Quast C. Pruesse E. Yilmaz P. Gerken J. Schweer T. Yarza P.. (2013). The SILVA ribosomal RNA gene database project: improved data processing and web-based tools. Nucleic Acids Res. 41, D590–D596. doi: 10.1093/nar/gks1219 23193283 PMC3531112

[B33] Roesch L. F. W. Dobbler P. T. Pylro V. S. Kolaczkowski B. Drew J. C. Triplett E. W. (2020). pime: A package for discovery of novel differences among microbial communities. Mol. Ecol. Resour. 20, 415–428. doi: 10.1111/1755-0998.13116 31698527

[B34] Rossi M. Martínez-Martínez D. Amaretti A. Ulrici A. Raimondi S. Moya A. (2016). Mining metagenomic whole genome sequences revealed subdominant but constant *Lactobacillus* population in the human gut microbiota. Environ. Microbiol. Rep. 8, 399–406. doi: 10.1111/1758-2229.12405 27043715

[B35] Russell J. T. Lauren Ruoss J. de la Cruz D. Li N. Bazacliu C. Patton L.. (2021). Antibiotics and the developing intestinal microbiome, metabolome and inflammatory environment in a randomized trial of preterm infants. Sci. Rep. 11, 1943. doi: 10.1038/s41598-021-80982-6 33479274 PMC7820285

[B36] Russell J. T. Roesch L. F. W. Ördberg M. Ilonen J. Atkinson M. A. Schatz D. A.. (2019). Genetic risk for autoimmunity is associated with distinct changes in the human gut microbiome. Nat. Commun. 10, 3621. doi: 10.1038/s41467-019-11460-x 31399563 PMC6689114

[B37] Schoonjans F. MedCalc Software Ltd. Odds ratio calculator. Available at: https://www.medcalc.org/calc/odds_ratio.php (Version 22.009) (accessed September 7, 2023).

[B38] Singh T. P. Tehri N. Kaur G. Malik R. K. (2021). Cell surface and extracellular proteins of potentially probiotic *Lactobacillus* reuteri as an effective mediator to regulate intestinal epithelial barrier function. Arch. Microbiol. 203, 3219–3228. doi: 10.1007/s00203-021-02318-2 33830286

[B39] Suez J. Zmora N. Elinav E. (2019). Probiotics in the next-generation sequencing era. Gut Microbes 11, 77–93. doi: 10.1080/19490976.2019.1586039 30951391 PMC6973336

[B40] Thomas N. J. Dennis J. M. Sharp S. A. Kaur A. Misra S. Walkey H. C.. (2021). DR15-DQ6 remains dominantly protective against type 1 diabetes throughout the first five decades of life. Diabetologia 64, 2258–2265. doi: 10.1007/s00125-021-05513-4 34272580 PMC8423681

[B41] Tinto N. Cola A. Piscopo C. Capuano M. Galatola M. Greco L.. (2015). High frequency of haplotype HLA-DQ7 in celiac disease patients from south Italy: retrospective evaluation of 5,535 subjects at risk of celiac disease. PloS One 10, e0138324. doi: 10.1371/journal.pone.0138324 26398634 PMC4580462

[B42] Vatanen T. Franzosa E. A. Schwager R. Tripathi S. Arthur T. D. Vehik K.. (2018). The human gut microbiome in early-onset type 1 diabetes from the TEDDY study. Nature 562, 589–594. doi: 10.1038/s41586-018-0620-2 30356183 PMC6296767

[B43] Wei T. Simko V. (2023). R package “corrplot”. In: Visualization of a correlation matrix. Available at: https://github.com/taiyun/corrplot (Accessed July 7, 2023).

[B44] Wickham H. (2016). ggplot2: elegant graphics for data analysis. Available at: https://ggplot2.tidyverse.org (Accessed July 6, 2022).

