# Supplementary material for: Human leukocyte antigen-dependent colonization of Lactobacillus in the early-life gut
Source: Front Microbiomes. 2023 Sep 29;2:1192773. doi: 10.3389/frmbi.2023.1192773 (PMC12993612; doi:10.3389/frmbi.2023.1192773)
Supplement: Supplementary file 1 [file DataSheet_1.zip › Supplementary Tables 1-3.DOCX]

Supplementary Material

Fleeting Colonization of Lactobacillus in the Early-Life Gut

Meghan A. Berryman^1^, Eric W. Triplett^1*^, Johnny Ludvigsson^2^

*** Correspondence:** Eric W. Triplett: ewt@ufl.edu

# Supplementary Tables

| **Supplemental Table 1. Number of infants in collection age groups.** | |
| --- | --- |
| **Age of Collection (months)** | **Number of Infants** |
| 0-1 | 8 |
| 1-2 | 3 |
| 2-3 | 5 |
| 3-4 | 12 |
| 4-5 | 25 |
| 5-6 | 51 |
| 6-7 | 16 |
| 7-8 | 11 |
| 8-9 | 3 |
| 9-10 | 39 |
| 10-11 | 203 |
| 11-12 | 271 |
| 12-13 | 485 |
| 13-14 | 245 |
| 14-15 | 64 |
| 15-16 | 36 |
| 16-17 | 13 |
| 17-18 | 11 |
| 18-X | 27 |

| **Supplemental Table 2. Read Identification by SILVA and comparison with NCBI BLAST.** Only the first entry from NCBI BLAST is included. | | | | |
| --- | --- | --- | --- | --- |
| **Format** | **Identity** | **Query Cover** | **Precent Identity** | **Accession Number** |
| SILVA | Lactobacillus casei 5909 | | | |
| BLAST | Lactobacillus casei strain IMAU60015 16S ribosomal RNA gene | 100% | 100% | FJ749748.1 |
| ASV | TAGGGAATCTTCCACAATGGACGCAAGTCTGATGGAGCAACGCCGCGTGAGTGAAGAAGGCTTTCGGGTCGTAAAACTCTGTTGTTGGAGAAGAATGGTCGGCAGAGTAACTGCTGCCGGCGTGACGGTATCCAACCAGAAAGCCACGGCTAACTACGTGCCAGCAGCCGCGGTAATACGTAGGTGGCAAGCGTTATCCGGATTTATTGGGCGTAAAGCGAGCGCAGGCGGTTTTTTAAGTCTGATGTGAAAGCCCTCGGCTTAACCGAGGAAGCGCATCGGAAACTGGGAAACTTGAGTGCAGAAGAGGACAGTGGAACTCCATGTGTAGCGGTGAAATGCGTAGATATATGGAAGAACACCAGTGGCGAAGGCGGCTGTCTGGTCTGTAACTGACGCTGAGGCTCGAAAGCATGGGTAGCGAACAGG | | | |
| SILVA | Lactobacillus delbrueckii 5913 |  |  |  |
| BLAST | Lactobacillus delbrueckii susp. bulgaricus strain 4040 16S ribosomal RNA gene, partial sequence | 100% | 100% | MT645507.1 |
| ASV | TAGGGAATCTTCCACAATGGACGCAAGTCTGATGGAGCAACGCCGCGTGAGTGAAGAAGGTTTTCGGATCGTAAAGCTCTGTTGTTGGTGAAGAAGGATAGAGGCAGTAACTGGTCTTTATTTGACGGTAATCAACCAGAAAGTCACGGCTAACTACGTGCCAGCAGCCGCGGTAATACGTAGGTGGCAAGCGTTGTCCGGATTTATTGGGCGTAAAGCGAGCGCAGGCGGAATGATAAGTCTGATGTGAAAGCCCACGGCTCAACCGTGGAACTGCATCGGAAACTGTCATTCTTGAGTGCAGAAGAGGAGAGTGGAATTCCATGTGTAGCGGTGGAATGCGTAGATATATGGAAGAACACCAGTGGCGAAGGCGGCTCTCTGGTCTGCAACTGACGCTGAGGCTCGAAAGCATGGGTAGCGAACAGG | | | |
| SILVA | Lactobacillus NA 5943 |  |  |  |
| BLAST | Lactoplantibacillus plantarum strain JCM 1149 16 ribosomal RNA, partial sequence | 100% | 100% | NR 115605.1 |
| ASV | TAGGGAATCTTCCACAATGGACGAAAGTCTGATGGAGCAACGCCGCGTGAGTGAAGAAGGGTTTCGGCTCGTAAAACTCTGTTGTTAAAGAAGAACATATCTGAGAGTAACTGTTCAGGTATTGACGGTATTTAACCAGAAAGCCACGGCTAACTACGTGCCAGCAGCCGCGGTAATACGTAGGTGGCAAGCGTTGTCCGGATTTATTGGGCGTAAAGCGAGCGCAGGCGGTTTTTTAAGTCTGATGTGAAAGCCTTCGGCTCAACCGAAGAAGTGCATCGGAAACTGGGAAACTTGAGTGCAGAAGAGGACAGTGGAACTCCATGTGTAGCGGTGAAATGCGTAGATATATGGAAGAACACCAGTGGCGAAGGCGGCTGTCTGGTCTGTAACTGACGCTGAGGCTCGAAAGTATGGGTAGCAAACAGG | | | |
| SILVA | Lactobacillus NA 5948 |  |  |  |
| BLAST | Latilactobacillus graminis strain LMG 9825 16S ribosomal RNA, partial sequence | 100% | 99.7% | NR 114916.1 |
| ASV | TAGGGAATCTTCCACAATGGACGAAAGTCTGATGGAGCAACGCCGCGTGAGTGAAGAAGGTTTTCGGATCGTAAAACTCTGTTGTTGGAGAAGAACGTATTTGATAGTAACTGATCAGGTAGTGACGGTATCCAACCAGAAAGCCACGGCTAACTACGTGCCAGCAGCCGCGGTAATACGTAGGTGGCAAGCGTTGTCCGGATTTATTGGGCGTAAAGCGAGCGCAGGCGGTTTCTTAAGTCTGATGTGAAAGCCTTCGGCTCAACCGAAGAAGTGCATCGGAAACTGGGAAACTTGAGTGCAGAAGAGGACAGTGGAACTCCATGTGTAGCGGTGAAATGCGTAGATATATGGAAGAACACCAGTGGCGAAGGCGGCTGTCTGGTCTGTAACTGACGCTGAGGCTCGAAAGCATGGGTAGCAAACAGG | | | |
| SILVA | Lactobacillus NA 5951 |  |  |  |
| BLAST | Latilactobacillus sakei strain NBRC 15893 16S ribosomal RNA, partial sequence | 100% | 100% | NR 113821.1 |
| ASV | TAGGGAATCTTCCACAATGGACGAAAGTCTGATGGAGCAACGCCGCGTGAGTGAAGAAGGTTTTCGGATCGTAAAACTCTGTTGTTGGAGAAGAATGTATCTGATAGTAACTGATCAGGTAGTGACGGTATCCAACCAGAAAGCCACGGCTAACTACGTGCCAGCAGCCGCGGTAATACGTAGGTGGCAAGCGTTGTCCGGATTTATTGGGCGTAAAGCGAGCGCAGGCGGTTTCTTAAGTCTGATGTGAAAGCCTTCGGCTCAACCGAAGAAGTGCATCGGAAACTGGGAAACTTGAGTGCAGAAGAGGACAGTGGAACTCCATGTGTAGCGGTGAAATGCGTAGATATATGGAAGAACACCAGTGGCGAAGGCGGCTGTCTGGTCTGTAACTGACGCTGAGGCTCGAAAGCATGGGTAGCAAACAGG | | | |
| SILVA | Lactobacillus NA 5952 |  |  |  |
| BLAST | Lactobacillus acidophilus strain VPI 6032 16S ribosomal RNA, partial sequence | 100% | 100% | NR 117062.1 |
| ASV | TAGGGAATCTTCCACAATGGACGAAAGTCTGATGGAGCAACGCCGCGTGAGTGAAGAAGGTTTTCGGATCGTAAAGCTCTGTTGTTGGTGAAGAAGGATAGAGGTAGTAACTGGCCTTTATTTGACGGTAATCAACCAGAAAGTCACGGCTAACTACGTGCCAGCAGCCGCGGTAATACGTAGGTGGCAAGCGTTGTCCGGATTTATTGGGCGTAAAGCGAGCGCAGGCGGAAGAATAAGTCTGATGTGAAAGCCCTCGGCTTAACCGAGGAACTGCATCGGAAACTGTTTTTCTTGAGTGCAGAAGAGGAGAGTGGAACTCCATGTGTAGCGGTGGAATGCGTAGATATATGGAAGAACACCAGTGGCGAAGGCGGCTCTCTGGTCTGCAACTGACGCTGAGGCTCGAAAGCATGGGTAGCGAACAGG | | | |
| SILVA | Lactobacillus NA 5954 |  |  |  |
| BLAST | Lentilactobacillus buchneri strain JCM 1115 16S ribosomal RNA, partial sequence | 100% | 100% | NR 041293.1 |
| ASV | TAGGGAATCTTCCACAATGGACGAAAGTCTGATGGAGCAACGCCGCGTGAGTGATGAAGGGTTTCGGCTCGTAAAACTCTGTTGTTGGAGAAGAACAGGTGTCAGAGTAACTGTTGACATCTTGACGGTATCCAACCAGAAAGCCACGGCTAACTACGTGCCAGCAGCCGCGGTAATACGTAGGTGGCAAGCGTTGTCCGGATTTATTGGGCGTAAAGCGAGCGCAGGCGGTTTTTTAGGTCTGATGTGAAAGCCTTCGGCTTAACCGGAGAAGTGCATCGGAAACCGGGAGACTTGAGTGCAGAAGAGGACAGTGGAACTCCATGTGTAGCGGTGAAATGCGTAGATATATGGAAGAACACCAGTGGCGAAGGCGGCTGTCTGGTCTGTAACTGACGCTGAGGCTCGAAAGCATGGGTAGCGAACAGG | | | |
| SILVA | Lactobacillus NA 5955 |  |  |  |
| BLAST | Lentilactobacillus parabuchneri strain DSM 5707 16S ribosomal RNA, partial sequence | 100% | 100% | NR 112755.1 |
| ASV | TAGGGAATCTTCCACAATGGACGAAAGTCTGATGGAGCAACGCCGCGTGAGTGATGAAGGGTTTCGGCTCGTAAAACTCTGTTGTTGGAGAAGAACAGGTGTGAGAGTAACTGTTCACATCTTGACGGTATCCAACCAGAAAGCCACGGCTAACTACGTGCCAGCAGCCGCGGTAATACGTAGGTGGCAAGCGTTGTCCGGATTTATTGGGCGTAAAGCGAGCGCAGGCGGTTTCTTAGGTCTGATGTGAAAGCCTTCGGCTTAACCGGAGAAGTGCATCGGAAACCAGGAGACTTGAGTGCAGAAGAGGACAGTGGAACTCCATGTGTAGCGGTGAAATGCGTAGATATATGGAAGAACACCAGTGGCGAAGGCGGCTGTCTGGTCTGTAACTGACGCTGAGGCTCGAAAGCATGGGTAGCGAACAGG | | | |
| SILVA | Lactobacillus NA 5958 |  |  |  |
| BLAST | Levilactobacillus brevis ATCC 14869 = DSM 20054 16S ribosomal RNA, partial sequence | 100% | 100% | NR 116238.1 |
| ASV | TAGGGAATCTTCCACAATGGACGAAAGTCTGATGGAGCAATGCCGCGTGAGTGAAGAAGGGTTTCGGCTCGTAAAACTCTGTTGTTAAAGAAGAACACCTTTGAGAGTAACTGTTCAAGGGTTGACGGTATTTAACCAGAAAGCCACGGCTAACTACGTGCCAGCAGCCGCGGTAATACGTAGGTGGCAAGCGTTGTCCGGATTTATTGGGCGTAAAGCGAGCGCAGGCGGTTTTTTAAGTCTGATGTGAAAGCCTTCGGCTTAACCGGAGAAGTGCATCGGAAACTGGGAGACTTGAGTGCAGAAGAGGACAGTGGAACTCCATGTGTAGCGGTGGAATGCGTAGATATATGGAAGAACACCAGTGGCGAAGGCGGCTGTCTAGTCTGTAACTGACGCTGAGGCTCGAAAGCATGGGTAGCGAACAGG | | | |
| SILVA | Lactobacillus NA 5961 |  |  |  |
| BLAST | Companilactobacillus alimentarius strain HBUAS66085 16S ribosomal RNA gene, partial sequence | 100% | 100% | OQ701480.1 |
| ASV | TAGGGAATCTTCCACAATGGACGAAAGTCTGATGGAGCAATGCCGCGTGAGTGAAGAAGGTTTTCGGATCGTAAAACTCTGTTGTTGAAGAAGAACATATGTGAGAGTAACTGTTCACGTACTGACGGTATTCAACCAGAAAGCCACGGCTAACTACGTGCCAGCAGCCGCGGTAATACGTAGGTGGCAAGCGTTGTCCGGATTTATTGGGCGTAAAGAGAATGTAGGCGGTTCATTAAGTTTGAAGTGAAAGCCCTCGGCTCAACCGAGGAAGTGCTTCGAAAACTGGTGAACTTGAGTGCAGAAGAGGAAAGTGGAACTCCATGTGTAGCGGTGGAATGCGTAGATATATGGAAGAACACCAGTGGCGAAGGCGGCTTTCTGGTCTGTAACTGACGCTGAGATTCGAAAGCATGGGTAGCAAACAGG | | | |
| SILVA | Lactobacillus NA 5968 |  |  |  |
| BLAST | Lacticaseibacillus paracasei strain NBRC 15889 16S ribosomal RNA, partial sequence | 100% | 99.77% | NR 113337.1 |
| ASV | TAGGGAATCTTCCACAATGGACGCAAGTCTGATGGAGCAACGCCGCGTGAGTGAAGAAGGCTTTCGGGTCGTAAAACTCTGTTGTTGGAGAAGAATGGTCGGCAGAGTAACTGTTGCCGGCGTGACGGTATCCAACCAGAAAGCCACGGCTAACTACGTGCCAGCAGCCGCGGTAATACGTAGGTGGCAAGCGTTATCCGGATTTATTGGGCGTAAAGCGAGCGCAGGCGGTTTTTTAAGTCTGATGTGAAAGCCCTCGGCTTAACCGAGGAAGCGCATCGGAAACTGGGAAACTTGAGTGCAGAAGAGGACAGTGGAACTCCATGTGTAGCGGTGAAATGCGTAGATATATGGAAGAACACCAGTGGCGAAGGCGGCTGTCTGGTCTGTAACTGACGCTGAGGCTCGAAAGCATGGGTAGCGAACAGG | | | |
| SILVA | Lactobacillus NA 5971 |  |  |  |
| BLAST | Lacticaseibacillus rhamnosus strain NBRC 3425 16S ribosomal RNA, partial sequence | 100% | 100% | NR 113332.1 |
| ASV | TAGGGAATCTTCCACAATGGACGCAAGTCTGATGGAGCAACGCCGCGTGAGTGAAGAAGGCTTTCGGGTCGTAAAACTCTGTTGTTGGAGAAGAATGGTCGGCAGAGTAACTGTTGTCGGCGTGACGGTATCCAACCAGAAAGCCACGGCTAACTACGTGCCAGCAGCCGCGGTAATACGTAGGTGGCAAGCGTTATCCGGATTTATTGGGCGTAAAGCGAGCGCAGGCGGTTTTTTAAGTCTGATGTGAAAGCCCTCGGCTTAACCGAGGAAGTGCATCGGAAACTGGGAAACTTGAGTGCAGAAGAGGACAGTGGAACTCCATGTGTAGCGGTGAAATGCGTAGATATATGGAAGAACACCAGTGGCGAAGGCGGCTGTCTGGTCTGTAACTGACGCTGAGGCTCGAAAGCATGGGTAGCGAACAGG | | | |
| SILVA | Lactobacillus NA 5972 |  |  |  |
| BLAST | Lacticaseibacillus paracasei strain NBRC 15883 16S ribosomal RNA, partial sequence | 100% | 100% | NR 113337.1 |
| ASV | TAGGGAATCTTCCACAATGGACGCAAGTCTGATGGAGCAACGCCGCGTGAGTGAAGAAGGCTTTCGGGTCGTAAAACTCTGTTGTTGGAGAAGAATGGTCGGCAGAGTAACTGTTGTCGGCGTGACGGTATCCAACCAGAAAGCCACGGCTAACTACGTGCCAGCAGCCGCGGTAATACGTAGGTGGCAAGCGTTATCCGGATTTATTGGGCGTAAAGCGAGCGCAGGCGGTTTTTTAAGTCTGATGTGAAAGCCCTCGGCTTAACCGAGGAAGCGCATCGGAAACTGGGAAACTTGAGTGCAGAAGAGGACAGTGGAACTCCATGTGTAGCGGTGAAATGCGTAGATATATGGAAGAACACCAGTGGCGAAGGCGGCTGTCTGGTCTGTAACTGACGCTGAGGCTCGAAAGCATGGGTAGCGAACAGG | | | |
| SILVA | Lactobacillus NA 5973 |  |  |  |
| BLAST | Lacticaseibacillus rhamnosus strain NBRC 3425 16S ribosomal RNA, partial sequence | 100% | 99.77% | NR 113332.1 |
| ASV | TAGGGAATCTTCCACAATGGACGCAAGTCTGATGGAGCAACGCCGCGTGAGTGAAGAAGGCTTTCGGGTCGTAAAACTCTGTTGTTGGAGAAGAATGGTCGGCAGAGTAACTGTTGTCGGCGTGACGGTATCCAACCAGAAAGCCACGGCTAACTACGTGCCAGCAGCCGCGGTAATACGTAGGTGGCAAGCGTTATCCGGATTTATTGGGCGTAAAGCGAGCGCAGGCGGTTTTTTAAGTCTGATGTGAAAGCCCTCGGCTTAACCGAGGAAGTGCATCGGAAACTGGAAAACTTGAGTGCAGAAGAGGACAGTGGAACTCCATGTGTAGCGGTGAAATGCGTAGATATATGGAAGAACACCAGTGGCGAAGGCGGCTGTCTGGTCTGTAACTGACGCTGAGGCTCGAAAGCATGGGTAGCGAACAGG | | | |
| SILVA | Lactobacillus NA 5981 |  |  |  |
| BLAST | Lactobacillus gasseri strain CIP 102991 16S ribosomal RNA, partial sequence | 100% | 100% | NR 117573.1 |
| ASV | TAGGGAATCTTCCACAATGGACGCAAGTCTGATGGAGCAACGCCGCGTGAGTGAAGAAGGGTTTCGGCTCGTAAAGCTCTGTTGGTAGTGAAGAAAGATAGAGGTAGTAACTGGCCTTTATTTGACGGTAATTACTTAGAAAGTCACGGCTAACTACGTGCCAGCAGCCGCGGTAATACGTAGGTGGCAAGCGTTGTCCGGATTTATTGGGCGTAAAGCGAGTGCAGGCGGTTCAATAAGTCTGATGTGAAAGCCTTCGGCTCAACCGGAGAATTGCATCAGAAACTGTTGAACTTGAGTGCAGAAGAGGAGAGTGGAACTCCATGTGTAGCGGTGGAATGCGTAGATATATGGAAGAACACCAGTGGCGAAGGCGGCTCTCTGGTCTGCAACTGACGCTGAGGCTCGAAAGCATGGGTAGCGAACAGG | | | |
| SILVA | Lactobacillus NA 5987 |  |  |  |
| BLAST | Ligilactobacillus salivarius strain HO 66 16S ribosomal RNA, partial sequence | 100% | 100% | NR 028725.2 |
| ASV | TAGGGAATCTTCCACAATGGACGCAAGTCTGATGGAGCAACGCCGCGTGAGTGAAGAAGGTCTTCGGATCGTAAAACTCTGTTGTTAGAGAAGAACACGAGTGAGAGTAACTGTTCATTCGATGACGGTATCTAACCAGCAAGTCACGGCTAACTACGTGCCAGCAGCCGCGGTAATACGTAGGTGGCAAGCGTTGTCCGGATTTATTGGGCGTAAAGGGAACGCAGGCGGTCTTTTAAGTCTGATGTGAAAGCCTTCGGCTTAACCGGAGTAGTGCATTGGAAACTGGAAGACTTGAGTGCAGAAGAGGAGAGTGGAACTCCATGTGTAGCGGTGAAATGCGTAGATATATGGAAGAACACCAGTGGCGAAAGCGGCTCTCTGGTCTGTAACTGACGCTGAGGTTCGAAAGCGTGGGTAGCAAACAGG | | | |
| SILVA | Lactobacillus NA 6008 |  |  |  |
| BLAST | Limosilactobacillus fermentum strain NBRC 15885 16S ribosomal RNA, partial sequence | 100% | 100% | NR 113335.1 |
| ASV | TAGGGAATCTTCCACAATGGGCGCAAGCCTGATGGAGCAACACCGCGTGAGTGAAGAAGGGTTTCGGCTCGTAAAGCTCTGTTGTTAAAGAAGAACACGTATGAGAGTAACTGTTCATACGTTGACGGTATTTAACCAGAAAGTCACGGCTAACTACGTGCCAGCAGCCGCGGTAATACGTAGGTGGCAAGCGTTATCCGGATTTATTGGGCGTAAAGAGAGTGCAGGCGGTTTTCTAAGTCTGATGTGAAAGCCTTCGGCTTAACCGGAGAAGTGCATCGGAAACTGGATAACTTGAGTGCAGAAGAGGGTAGTGGAACTCCATGTGTAGCGGTGGAATGCGTAGATATATGGAAGAACACCAGTGGCGAAGGCGGCTACCTGGTCTGCAACTGACGCTGAGACTCGAAAGCATGGGTAGCGAACAGG | | | |
| SILVA | Lactobacillus NA 6013 |  |  |  |
| BLAST | Limosilactobacillus mucosae S32 16S ribosomal RNA, partial sequence | 100% | 100% | NR 024994.1 |
| ASV | TAGGGAATCTTCCACAATGGGCGCAAGCCTGATGGAGCAACACCGCGTGAGTGAAGAAGGGTTTCGGCTCGTAAAGCTCTGTTGTTAGAGAAGAACGTGCGTGAGAGCAACTGTTCACGCAGTGACGGTATCTAACCAGAAAGTCACGGCTAACTACGTGCCAGCAGCCGCGGTAATACGTAGGTGGCAAGCGTTATCCGGATTTATTGGGCGTAAAGCGAGCGCAGGCGGTTTGATAAGTCTGATGTGAAAGCCTTTGGCTTAACCAAAGAAGTGCATCGGAAACTGTCAGACTTGAGTGCAGAAGAGGACAGTGGAACTCCATGTGTAGCGGTGGAATGCGTAGATATATGGAAGAACACCAGTGGCGAAGGCGGCTGTCTGGTCTGCAACTGACGCTGAGGCTCGAAAGCATGGGTAGCGAACAGG | | | |
| SILVA | Lactobacillus NA 6020 |  |  |  |
| BLAST | Limosilactobacillus reuteri subsp. reuteri strain DSM 20016 16S ribosomal RNA, partial sequence | 100% | 100% | NR 075036.1 |
| ASV | TAGGGAATCTTCCACAATGGGCGCAAGCCTGATGGAGCAACACCGCGTGAGTGAAGAAGGGTTTCGGCTCGTAAAGCTCTGTTGTTGGAGAAGAACGTGCGTGAGAGTAACTGTTCACGCAGTGACGGTATCCAACCAGAAAGTCACGGCTAACTACGTGCCAGCAGCCGCGGTAATACGTAGGTGGCAAGCGTTATCCGGATTTATTGGGCGTAAAGCGAGCGCAGGCGGTTGCTTAGGTCTGATGTGAAAGCCTTCGGCTTAACCGAAGAAGTGCATCGGAAACCGGGCGACTTGAGTGCAGAAGAGGACAGTGGAACTCCATGTGTAGCGGTGGAATGCGTAGATATATGGAAGAACACCAGTGGCGAAGGCGGCTGTCTGGTCTGCAACTGACGCTGAGGCTCGAAAGCATGGGTAGCGAACAGG | | | |
| SILVA | Lactobacillus oris 6028 |  |  |  |
| BLAST | Limosilactobacillus oris strain DSM 4864 16S ribosomal RNA, partial sequence | 100% | 100% | NR 026309.1 |
| ASV | TAGGGAATCTTCCACAATGGGCGCAAGCCTGATGGAGCAACACCGCGTGAGTGAAGAAGGGTTTCGGCTCGTAAAACTCTGTTGTTGGAGAAGAACGTGCGTAAGAGTAACTGTTTACGCAGTGACGGTATCCAACCAGAAAGTCACGGCTAACTACGTGCCAGCAGCCGCGGTAATACGTAGGTGGCAAGCGTTATCCGGATTTATTGGGCGTAAAGCGAGCGCAGGCGGTTGCTTAGGTCTGATGTGAAAGCCTTCGGCTTAACCGAAGAAGTGCATCGGAAACCGGGCGACTTGAGTGCAGAAGAGGACAGTGGAACTCCATGTGTAGCGGTGGAATGCGTAGATATATGGAAGAACACCAGTGGCGAAGGCGGCTGTCTGGTCTGCAACTGACGCTGAGGCTCGAAAGCATGGGTAGCGAACAGG | | | |
| SILVA | Lactobacillus reuteri 6038 |  |  |  |
| BLAST | Limosilactobacillus reuteri subsp. reuteri strain DSM 20016 16S ribosomal RNA, partial sequence | 100% | 99.30% | NR 075036.1 |
| ASV | TAGGGAATCTTCCACAATGGGCGCAAGCCTGATGGAGCAACACCGCGTGAGTGAAGAAGGGTTTCGGCTCGTAAAGCTCTGTTGTTGGAGAAGAATATGCGTGAGAGTAACTGTTCACGCAGTGACGGTATCCAACCAGAAAGTCACGGCTAACTACGTGCCAGCAGCCGCGGTAATACGTAGGTGGCAAGCGTTATCCGGATTTATTGGGCGTAAAGCGAGCGCAGGCGGTTGCTTAGGTCTGATGTGAAAGCCTTCGGCTTAACCGAAGAAGTGCATCGGAAACCGGGCGACTTGAGTGCAGAAGAGGACAGTGGAACTCCATGTGTAGCGGTGGAATGCGTAGATATATGGAAGAACACCAGTGGCGAAGGCGACTGTCTGGTCTGCAACTGACGCTGAGGCTCGAAAGCATGGGTAGCGAACAGG | | | |
| SILVA | Lactobacillus rhamnosus 6039 |  |  |  |
| BLAST | Lacticaseibacillus rhamnosus strain NBRC 3425 16S ribosomal RNA, partial sequence | 100% | 99.77% | NR 113332.1 |
| ASV | TAGGGAATCTTCCACAATGGACGCAAGTCTGATGGAGCAACGCCGCGTGAGTGAAGAAGGCTTTCGGGTCGTAAAACTCTGTTGTTGGAGAAGAATGGTCGGCAGAGTAACTGTTGTCGGCGTGACGGTATCCAACCAGAAAGCCACGGCTAACTACGTGCCAGCAGCCGCGGTAATACGTAGGTGGCAAGCGTTATCCGGATTTATTGGGCGTAAAGCGAGCGCAGGCGGTTTTTTAAGTCTGATGTGAAAGCCCTCGGCTTAACCGAGGAAGTGCATCGGAAACTGGGAAACTTGAGTGCAGAAAAGGACAGTGGAACTCCATGTGTAGCGGTGAAATGCGTAGATATATGGAAGAACACCAGTGGCGAAGGCGGCTGTCTGGTCTGTAACTGACGCTGAGGCTCGAAAGCATGGGTAGCGAACAGG | | | |
| SILVA | Lactobacillus rhamnosus 6040 |  |  |  |
| BLAST | Lacticaseibacillus rhamnosus strain NBRC 3425 16S ribosomal RNA, partial sequence | 100% | 99.77% | NR 113332.1 |
| ASV | TAGGGAATCTTCCACAATGGACGCAAGTCTGATGGAGCAACGCCGCGTGAGTGAAGAAGGCTTTCGGGTCGTAAAACTCTGTTGTTGGAGAAGAATGGTCGGCAGAGTAACTGTTGTCGGCGTGACGGTATCCAACCAGAAAGCCACGGCTAACTACGTGCCAGCAGCCGCGGTAATACGTAGGTGGCAAGCGTTATCCGGATTTATTGGGCGTAAAGCGAGCGCAGGCGGTTTTTTAAGTCTGATGTGAAAGCCCTCGGCTTAACCGAGGAAGTGCATCGGAAACTGGGAAACTTGAGTACAGAAGAGGACAGTGGAACTCCATGTGTAGCGGTGAAATGCGTAGATATATGGAAGAACACCAGTGGCGAAGGCGGCTGTCTGGTCTGTAACTGACGCTGAGGCTCGAAAGCATGGGTAGCGAACAGG | | | |
| SILVA | Lactobacillus ruminis 6041 |  |  |  |
| BLAST | Ligilactobacillus ruminis strain NBRC 102161 16S ribosomal RNA, partial sequence | 100% | 99.77% | NR 041611.1 |
| ASV | TAGGGAATCTTCCACAATGGACGAAAGTCTGATGGAGCAACGCCGCGTGAATGAAGAAGGCCTTCGGGTCGTAAAATTCTGTTGTCAGAGAAGAACGTGCGTGAGAGTAACTGTTCACGTATTGACGGTATCTGACCAGAAAGCCACGGCTAACTACGTGCCAGCAGCCGCGGTAATACGTAGGTGGCAAGCGTTGTCCGGATTTATTGGGCGTAAAGGGAACGCAGGCGGTCTTTTAAGTCTGATGTGAAAGCCTTCGGCTTAACCGAAGTAGTGCATTGGAAACTGGAAGACTTGAGTGCAGAAGAGGAGAGTGGAACTCCATGTGTAGCGGTGAAATGCGTAGATATATGGAAGAACACCAGTGGCGAAAGCGGCTCTCTGGTCTGTAACTGACGCTGAGGTTCGAAAGCGTGGGTAGCAAACAGG | | | |
| SILVA | Lactobacillus ruminis 6042 |  |  |  |
| BLAST | Ligilactobacillus ruminis strain NBRC 102161 16S ribosomal RNA, partial sequence | 100% | 100% | NR 041611.1 |
| ASV | TAGGGAATCTTCCACAATGGACGAAAGTCTGATGGAGCAACGCCGCGTGAATGAAGAAGGCCTTCGGGTCGTAAAATTCTGTTGTCAGAGAAGAACGTGCGTGAGAGTAACTGTTCACGTATTGACGGTATCTGACCAGAAAGCCACGGCTAACTACGTGCCAGCAGCCGCGGTAATACGTAGGTGGCGAGCGTTGTCCGGATTTATTGGGCGTAAAGGGAACGCAGGCGGTCTTTTAAGTCTGATGTGAAAGCCTTCGGCTTAACCGAAGTAGTGCATTGGAAACTGGAAGACTTGAGTGCAGAAGAGGAGAGTGGAACTCCATGTGTAGCGGTGAAATGCGTAGATATATGGAAGAACACCAGTGGCGAAAGCGGCTCTCTGGTCTGTAACTGACGCTGAGGTTCGAAAGCGTGGGTAGCAAACAGG | | | |

| **Supplemental Table 3. Significant associations between environmental confounders and *Lactobacillus* presence vs. absence.** Active breastfeeding: diet included breastmilk at the time of stool sample collection. Total months breastfeeding: duration of time the diet included any amount of breastmilk. Chi-squared test of independence. P-values adjusted for false discovery rate (FDR) using the Benjamini-Hochberg method. Only p_adj_ < 0.05 included. | | |
| --- | --- | --- |
| **Variable** | **Pvalue** | **FDR** |
| Active Breastfeeding | 5.00E-04 | 2.85E-02 |
| Total Months Breastfeeding  (Binned by 3 months) | 1.50E-03 | 4.27E-02 |
| Age by Month | 3.00E-03 | 4.56E-02 |
| Region | 3.50E-03 | 4.56E-02 |
| Introduction to Formula  (Binned by 6 months) | 4.00E-03 | 4.56E-02 |
